# A pilot *in vivo* study: potential ovarian cancer therapeutic by placental extracellular vesicles

**DOI:** 10.1042/BSR20230307

**Published:** 2023-08-18

**Authors:** Xinyue Chen, Sofian Tijono, Bridget Tsai, Lawrence William Chamley, Lai-Ming Ching, Qi Chen

**Affiliations:** 1 Department of Obstetrics and Gynaecology, Faculty of Medical and Health Science, The University of Auckland, Auckland, New Zealand; 2 Auckland Cancer Society Research Centre, The University of Auckland, Auckland, New Zealand

**Keywords:** CD169+, necrosis, ovarian cancer, placental EVs, tumour regression, tumour xenograft model

## Abstract

The biological links between cancer and pregnancy are of interest due to parallel proliferative, immunosuppressive, and invasive mechanisms between tumour and placental cells. However, the proliferation and invasion of placental cells are strictly regulated. The understanding of this regulation is largely unknown. Placental extracellular vesicles (EVs) may play an important role in this regulation, as placental EVs are known to contribute to maternal adaptation, including adaptation of the vascular and immune systems. We have previously reported that placental EVs significantly inhibited ovarian cancer cell proliferation by delaying the progression of the cell cycle. We, therefore, performed this pilot *in vivo* study to investigate whether placental EVs can also inhibit ovarian tumour growth in a SKOV-3 human tumour xenograft model. A single intraperitoneal injection of placental EVs at 15 days post tumour implantation, significantly inhibited the growth of the tumours in our *in vivo* model. Signs of cellular necrosis were observed in the ovarian tumour tissues, but not in other organs collected from mice that had been treated with placental EVs. Expression of receptor-interacting kinase 1 (RIPK1) and mixed linkage kinase domain-like (MLKL), which are mediators of necroptosis were not observed in our xenografted tumours. However, extensive infiltration of CD169^+^ macrophages and NK cells in ovarian tumour tissues collected from placental micro-EVs treated mice were observed. We demonstrate here that inhibition of ovarian tumour growth in our xenograft model by placental EVs involves cellular necrosis and infiltration of CD169^+^ macrophages and NK cells into the tumour tissues.

## Introduction

Ovarian cancer is a leading cause of death among all gynecological malignancies globally [1] and the survival rate is relatively low due to this cancer commonly being diagnosed at a later stage. Currently, the biological links between cancer and the placenta are of interest, due to parallel proliferative, immunosuppressive, and invasive mechanisms between tumour and placental cells (trophoblast). Although the mechanism of the strictly regulated proliferation and invasion of placental cells is largely unknown, a newly emerging physiological control system is, placental extracellular vesicles (EVs).

Like other EVs, placental EVs are lipid bilayer-enclosed packages of cellular contents that are involved in cell–cell communication and signalling [1]. It is well-known that during pregnancy, a large number of EVs are extruded from the placenta and are involved in the regulation of multiple maternal systems [2–7]. The underlying mechanisms of regulating maternal adaptation in pregnancy are due in part, to placental EVs being internalised by the target cells then releasing their regulatory cargos in the target cells; consequently impacting on the function of target cells [8]. Placental EVs carry pro-apoptotic proteins [9–12], and miRNAs, particularly those of the chromosome 19 microRNA cluster (C19MC) that are carried uniquely by placental EVs, which might attenuate growth of target cells [13].

We have previously shown that placental EVs, but not monocyte-derived control EVs, inhibited the *in vitro* proliferation of ovarian cancer cells [14]. Mechanistically this inhibition was due to delaying cell cycle progression [14]. Although the underlying mechanism of placental EVs delaying the ovarian cancer cell cycle is unknown, one study reported that placental nano-EVs containing C19MC miRNA could attenuate biological replication in target cells via autophagy-mediated pathways [13]. In addition, a recent study reported that miRNAs (miR-451a, miR-302d-3p and miR-223-3p) in EVs from the maternal circulation have a potential role in inhibiting cancer cell growth and metastasis, as well as promotion of cancer cell death [15]. Collectively, the regulatory activities of EVs are mainly mediated by proteins [14,16], and/or regulatory miRNAs that EVs carry [17].

Given the functions of placental EVs on the regulation of maternal adaptation during pregnancy and inhibition of ovarian cancer cell growth that we have previously reported, we undertook this pilot *in vivo* study to investigate whether placental EVs have anti-ovarian tumour growth activity and the potential underlying mechanism of this protective activity.

## Materials and methods

All animal experiments conformed to local institutional guidelines that meet the standards required by the UKCCCR guidelines and are under the declaration of Helsinki. This *in vivo* study was approved by the Animal Ethics Committee of the University of Auckland (AEC # 002257). The animal experiments took place at the University of Auckland, New Zealand. All animals were euthanized by cervical dislocation. The collection of human first trimester placenta with written informed consent has been approved by the Northern Regional Health & Disability Ethics Committee, Auckland, New Zealand (NTX/12/06/057/AM11).

### Collection of placental EVs

First trimester placentae were collected following elective surgical termination of on-going pregnancy at Epsom Day Unit, National Women’s Health, Auckland City Hospital, Auckland, New Zealand.

Placental micro- and nano-EVs were collected from placental explant cultures as previously described [18]. Briefly, placental explants (approximately 400 mg) were dissected and cultured in 12 well-plates with Netwell™ culture inserts (400 µm mesh) for 18 h at 37°C in Advanced DMEM/F12 containing 2% fetal bovine serum (FBS) in an ambient oxygen atmosphere containing 5% CO_2_. The conditioned media were collected and cellular debris removed by centrifuging at 2,000 × ***g*** for 10 min. Micro- and nano-EVs were then collected by centrifuging the supernatant at 20,000 × ***g*** for 1 h for micro-EVs collection. The supernatant was further centrifuged at 100,000 × ***g*** for 1 h for nano-EVs collection (Avanti J30I Ultracentrifuge, JA 30.50 fixed angle rotor, Beckman Coulter, New Zealand). The EVs were resuspended in PBS and protein levels were measured by BCA assay.

### SKOV-3 cell culture

The human ovarian adenocarcinoma SKOV-3 cell line from ATCC, was obtained courtesy of Dr Wouter van Leeuwen (ACSRC) and was maintained in αMEM (Gibco BRL, Grand Island, NY) supplemented with 5% FBS and antibiotics (100 U/ml penicillin and 100 μg/ml streptomycin) in a humidified incubator at 37°C with of 5% CO_2_.

### Mice and tumour implantations

Immunodeficient CD1 nude mice bred at the Vernon Jansen Unit, University of Auckland, were used as the hosts for the growth of human SKOV-3 tumours. Mice were inoculated subcutaneously into the left flank of each mouse with 10^6^ cells in 100 µl medium. Six days after inoculation, mice with tumours between 2 and 4 mm in diameter were randomized into three groups (*n* = 6 per group). The control group was injected intraperitoneally (ip) with 100 µl of PBS. Another group was injected with 100 µl placental-micro EVs and a third group was injected with 100 µl of placental-nano EV (100 µg of protein), respectively. Tumour size was measured thrice weekly, and tumour volumes were calculated as 0.52*a^2^b*, where *a* and *b* are the minor and major axes of the tumour. Mice were killed by cervical dislocation on day 58 after treatment (due to a lockdown caused by the COVID-19), and tumours, livers, and spleens were collected immediately and stored at −80°C until further processing for histological analyses.

### Organ dissection and sectioning

The tumour, liver and spleen tissues embedded in OCT tissue freezing medium were sectioned (5 μm) using a Cryostat (Cryostar NX70, ThermoFisher Scientific, Auckland, Newland). Slides were air-dried for 1 h and were stored at 4°C until needed for staining. The number of tumours large enough to use in this study was five for controls, and three for micro-EVs treated, and five for nano-EVs treated mice.

### Haematoxylin & Eosin (H&E) Staining

Slides were taken out from 4°C refrigerator and left for 15 min to warm to room temperature before use. After washing three times with PBS, the slides were stained with haematoxylin (Sigma-Aldrich, Auckland, New Zealand) for 15 min, washed three times with tap water and then the slides were stained with water soluble eosin (Sigma-Aldrich, Auckland, New Zealand) for 30 s. After washing three times with tap water, slides were covered with VectaMount permanent mounting medium (Vector Laboratories, Inc, Auckland, New Zealand) dried then examined using a light microscope (Nikon Digital Slight DS-vil Colour Microscope Camera, Nikon Corporation, Tokyo, Japan).

### Immunohistochemistry

Tissue sections at room temperature were fixed with 4% paraformaldehyde (PFA) for 15 min and rinsed three times with PBS. Slides were then incubated with blocking buffer (10% normal goat serum in 0.5% PBS-T) for 30 min. Primary antibodies, mouse anti-human total receptor-interacting kinase 1 (RIPK1) (1:200, VMA00421, Bio-Rad Laboratories, U.S.A.) or rabbit anti-human phospho-mixed linkage kinase domain-like (MLKL) (1:200, ThermoFisher, NZ) were then added for 2 h at room temperature. After washing three times with PBS-T, biotinylated secondary antibody from an ABC kit containing both anti-rabbit and anti-mouse secondary antibodies (Invitrogen, NZ) was added to each section and incubated for 10 min. After washing three times with PBS-T, AEC solution (Dako North America, Inc.) was then added and incubated for 5–10 min, depending on the colour development. After a final wash three times with PBS-T, sections were then counterstained for 6 min with haematoxylin and mounted with mounting medium (Vector Laboratories, U.S.A.).

### Immunofluorescence staining

Refrigerated slides were warmed to room temperature and then fixed with 4% paraformaldehyde (PFA) for 15 min and rinsed three times with PBS. Primary antibodies, rabbit anti-mouse CD45 antibody (1:200, Invitrogen, NZ), rabbit anti-mouse CD19 antibody (1:200, Biolegend), rabbit anti-mouse F4/80 antibody (1:200, Biolegend), rabbit anti-mouse CD169 antibody (1:200, Bio-Rad Laboratories, U.S.A.), or mouse anti-human NKp46/NKR1 antibody (1:100, Novus Biologicals, U.S.A.), were added for 18 h at 4°C. A secondary antibody with the fluorescent label (Alexa Fluor 488-AffiniPure Donkey Anti-Rat IgG, which reacts with both rabbit and mouse IgG, Jackson ImmunoResearch, United Kingdom) was added at 1:200 to the tissue sections and stained for 2 h at room temperature in a humidified chamber covered with aluminum foil. The secondary antibody solution was decanted, and the slides washed with buffer 3 times for 5 min each in the dark. Slides were then immersed in PBS in a Coplin jar for the final wash and then drained. A drop of mounting medium containing DAPI was added to the slides mounted with a coverslip.

### Semi-quantification and statistical analysis

The expression of CD169 and NKp46 in ovarian tumour tissues was semi-quantified using ImageJ. The fluorescent intensity collected from ImageJ was expressed as median and range. Mann–Whitney test was performed using the Prism software package (Version 9.3). The comparison of the tumour volume between the groups was assessed using unpaired *t*-test. *P*<0.05 was considered as significant.

## Results

### Placental EVs inhibited ovarian tumour growth

**Figure 1:**
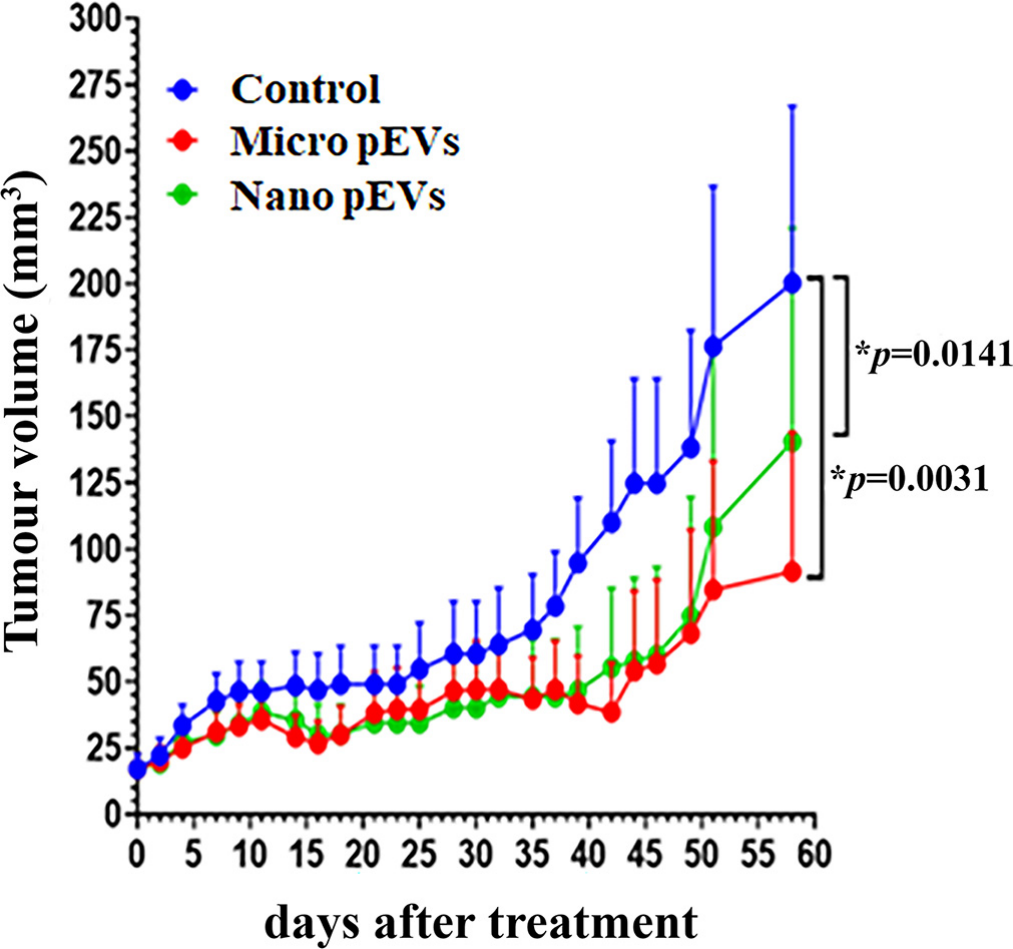
Growth chart of tumour The size of tumours in CD-1 nude mice that had been treated with placental micro-EVs (red line) and nano-EVs (green line) was significantly reduced, compared to control (blue line).

A single injection of placental micro- or nano-EVs on day 6 after implantation, caused a significant reduction in the size of SKOV-3 human tumour xenografts (*P*=0.0031 or *P*=0.0141, Figure 1), compared with tumours in the control group. A reduction of tumour size in placental EVs treated groups was observed up to 58 days after placental EV treatment when the mice were killed. Interestingly, a larger reduction of tumour volume was seen after 50 days, until 58 days in mice that had been treated with placental micro-EVs compared with those treated with nano-EVs.

### Evidence of necrosis in the ovarian tumour tissues from mice treated with placental EVs

**Figure 2:**
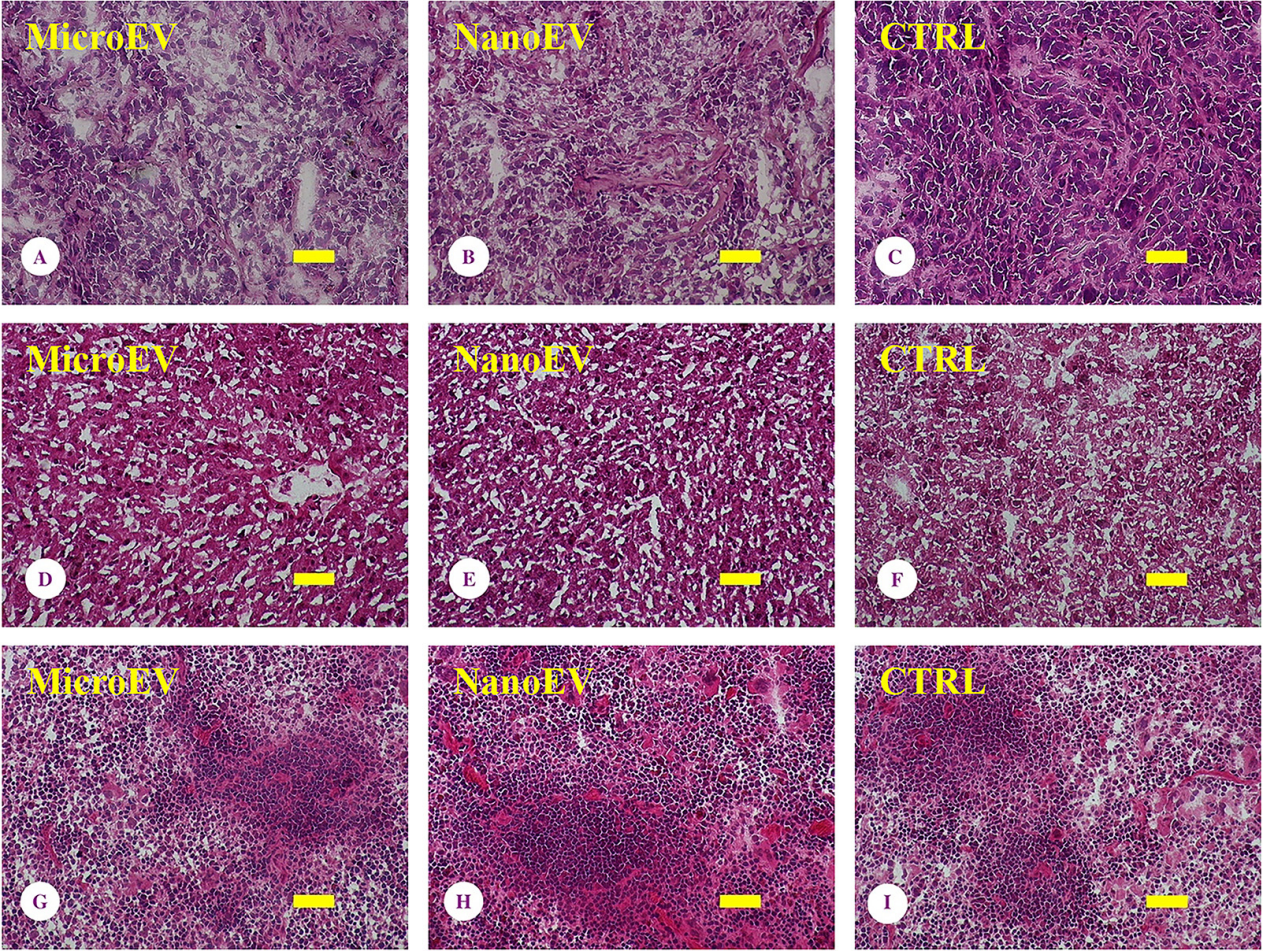
Histology examination Signs of necrosis were present in tumours (**A–C**), but not in liver (**D–F**) and spleen (**G–I**), collected from mice that had been treated with placental micro-EVs (A, D, G) and nano-EVs (B, E, I), compared with controls (C, F, I). Bar = 100 µm. The consistent finding was seen in tumour tissues collected from five untreated mice, and three micro-EVs treated, and five nano-EVs treated mice (Supplementary Figure S6).

To understand the mechanism that caused the reduction of ovarian tumour volume, the remaining SKOV-3 human tumour xenografts were examined histologically. As shown by H&E staining in Figure 2; although there were clear cellular outlines in the tumour tissue, there was a decrease in cellular volume and the presence of shrunken nuclei with condensed pigmentation. There was also an abundance of clear cytoplasm with large gaps between ovarian tumour cells seen in ovarian tumour tissues from mice that had been treated with placental micro- (Figure 2A) or nano-EVs (Figure 2B). In contrast, high cellular density with no clear space between tumour cells and enlargement, condensation or in irregular shaped nuclei were observed in ovarian tumour tissues collected from control mice (Figure 2C). The consistent finding was seen in tumour tissues collected from five untreated mice, and three micro-EVs treated, and five nano-EVs treated mice (Supplementary Figure S6).

To further investigate whether the histological changes seen in tumour tissues were also seen in non-tumour tissues, the histological sections of liver and spleens collected from the same mice were examined. Normal cellular integrity of hepatic (Figure 2D–F) and splenic cells (Figure 2G–I) with normal organ architecture was observed, and there was no difference in the appearance of cells in the livers or spleens collected from placental EV-treated and control mice. The consistent finding was seen in tumour tissues collected from five untreated mice, and three micro-EVs treated, and five nano-EVs treated mice.

### No expression of RIPK1 and MLKL in ovarian tumour tissues from mice that had been treated with placental EVs

**Figure 3:**
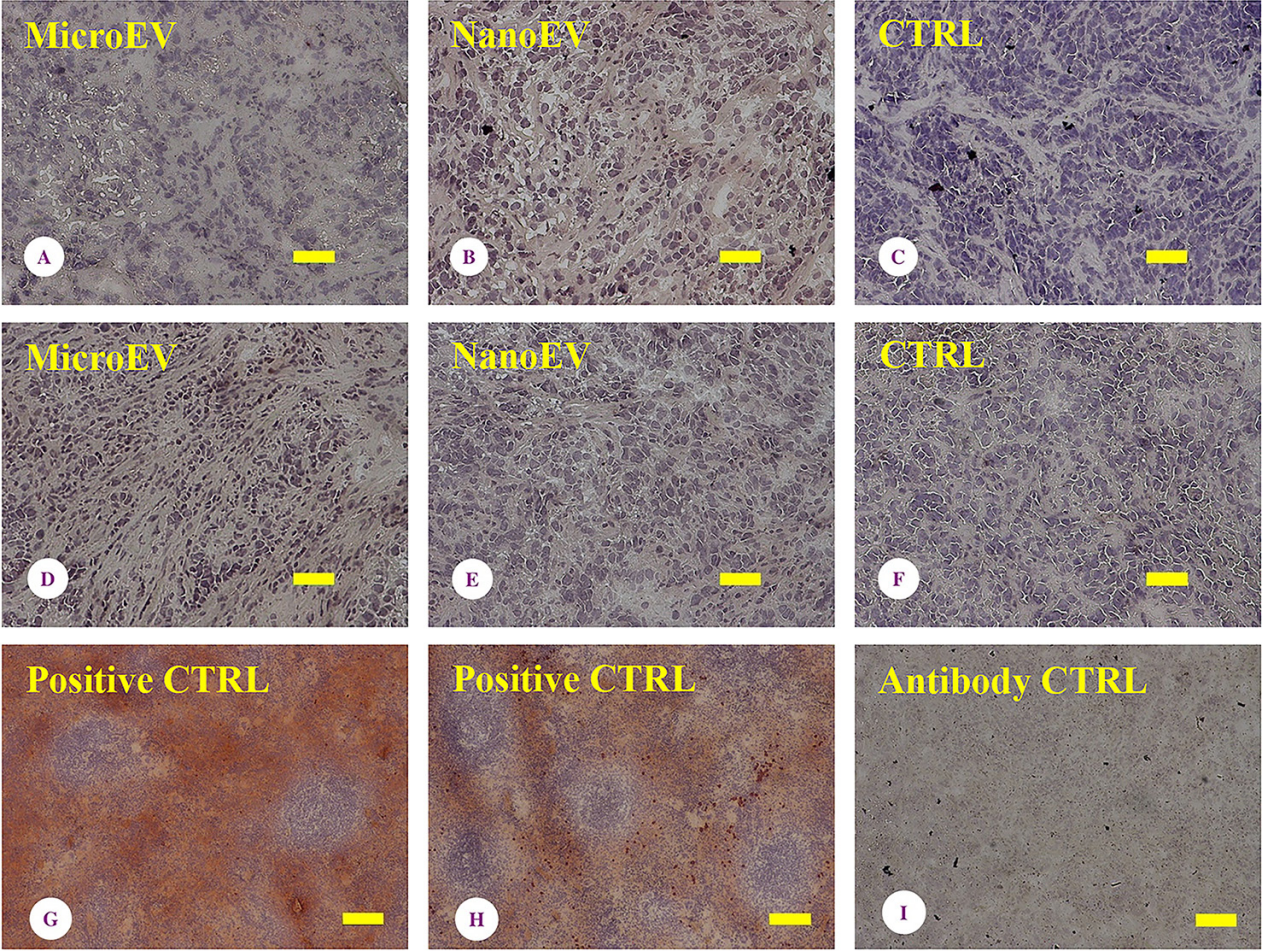
Analysis of apoptosis associated proteins No expression of RIPK1 (**A–C**) or MLKL (**D–F**) was present in tumours collected from mice that had been treated with placental micro-EVs (A,D) or nano-EVs (B,E), compared with untreated (C,F). Positive staining controls of spleen for the RIPK1 (**G**) and MLKL (**H**) antibodies. (**I**): antibody control. Bar = 100 µm. The consistent finding was seen in tumour tissues collected from five untreated mice, and three micro-EVs treated, and five nano-EVs treated mice

To further investigate the pathways triggering necrosis by placental EVs in ovarian tumour tissues, the expression of total RIPK1 and phospho-MLKL, which are mediators of necroptosis [19] were examined. As shown in Figure 3, there was no expression of RIPK1 (Figure 3A–C) or phospho-MLKL (Figure 3D–F) in ovarian tumour tissues collected from mice that had been treated with placental micro- or nano-EVs. The consistent finding was seen in tumour tissues collected from five untreated mice, and three micro-EVs treated, and five nano-EVs treated mice.

### No difference in the abundance of CD45 or F4/80 positive cells, and no detectable expression of CD19 in the ovarian tumour tissues among the treatment groups

Beneficial anti-cancer effects induced by immune cells have been well documented. We examined the abundance of CD45 (total leucocytes), F4/80 (monocyte/macrophages) and CD19 (B cells) in ovarian tumour tissues collected from mice that had been treated with placental micro- or nano-EVs. There was no difference in the number of CD45 (Supplementary Figure S1A–C) or F4/80 positive cells seen between ovarian tumour tissues from mice treated with placental micro- or nano-EVs Supplementary Figure S2A–C). In addition, there were no detectable CD19 positive cells in ovarian tumour tissues from any group (Supplementary Figure S3A–C). All these findings were observed in tumour tissues collected from four untreated mice, and three micro-EVs treated, and four nano-EVs treated mice.

### Increased CD169 positive macrophages and natural killer cells in ovarian tumour tissues from mice that had been treated with placental EVs

**Figure 4:**
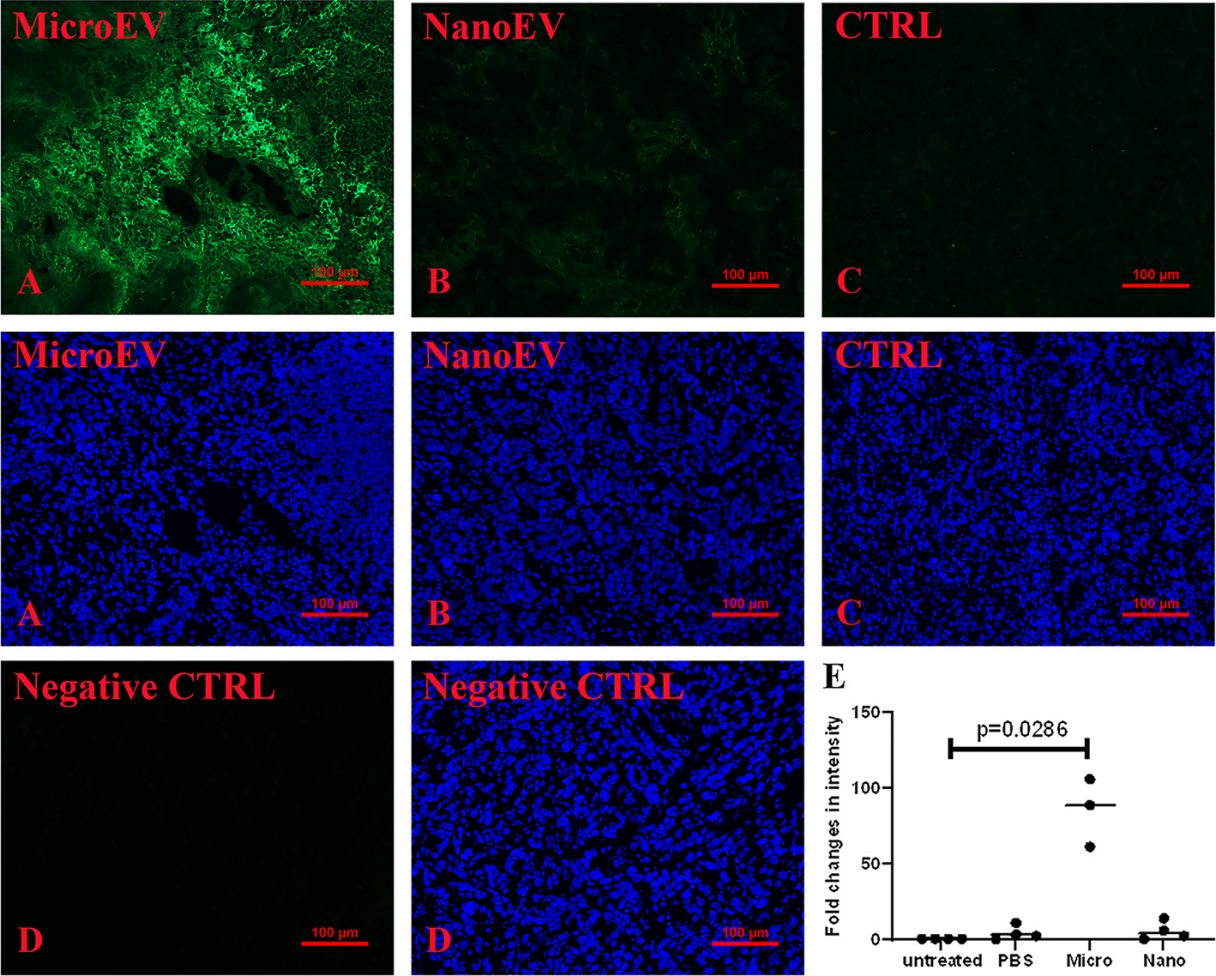
Immune response in tumour tissues Significantly higher intensity of CD169 was present in tumours collected from mice that had been treated with placental micro-EVs (**A**), compared with mice that had been treated with placental nano-EVs (**B**) or controls (**C**), measured by semi-quantitative analysis (**E**). (**D**): antibody control. The consistent finding was seen in tumour tissues collected from four untreated mice, and three micro-EVs treated, and four nano-EVs treated mice (Supplementary Figure S7).

There have been reports of higher fluorescence intensity of CD169^+^ macrophages being associated with a longer survival period and a good clinical prognosis in cancer patients [20–22]. The intensity of CD169^+^ macrophages in ovarian tumour tissues collected from the different treatment groups in this study is shown in Figure 4. Significantly higher fluorescence intensity of CD169^+^ macrophages was seen in ovarian tumour tissues collected from mice that had been treated with placental micro-EVs (Figure 4A), compared with those collected from mice treated with placental nano-EVs (Figure 4B) or control mice (Figure 4C), as determined by semi-quantitative analysis (Figure 4D, *P*=0.0286, Mann–Whitney test). The consistent finding was seen in tumour tissues collected from four untreated mice, and three micro-EVs treated, and four nano-EVs treated mice (Supplementary Figure S7). There was no significant difference in the fluorescence intensity of CD169^+^ macrophages in the livers (Supplementary Figure S4A–C) or spleens collected from different treatment groups (Supplementary Figure S5A–C).

**Figure 5:**
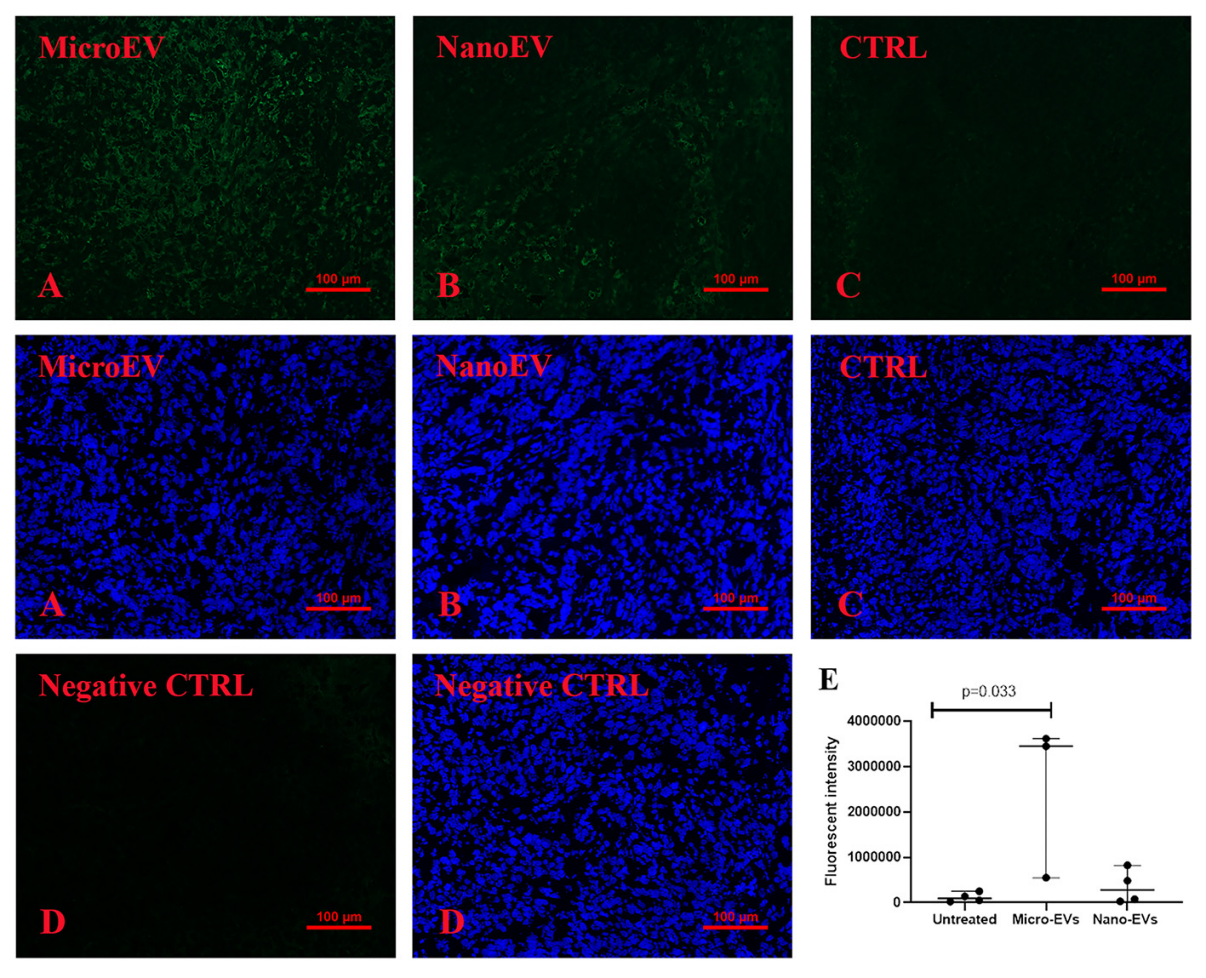
Immune response in tumour tissues Significantly higher intensity of NKp46 was present in tumours collected from mice that had been treated with placental micro-EVs (**A**), compared with mice that had been treated with placental nano-EVs (**B**) or controls (**C**), measured by semi-quantitative analysis (**E**). (**D**): antibody control. The consistent finding was seen in tumour tissues collected from four untreated mice, and three micro-EVs treated, and four nano-EVs treated mice (Supplementary Figure S8).

Natural killer (NK) cells have been suggested to have potent antitumour activity [23], and the expression of NKp46, as a marker of murine NK cells was examined. A significantly higher expression of NKp46 was seen in ovarian tumour tissues collected from mice that had been treated with placental micro-EVs (Figure 5A), compared with those treated with placental nano-EVs (Figure 5B) and controls (Figure 5C,D, *P*=0.033, Mann–Whitney test). The consistent finding was seen in tumour tissues collected from four untreated mice, and three micro-EVs treated, and four nano-EVs treated mice (Supplementary Figure S8).

The correlation of fluorescence intensity of CD169 and NKp46 in ovarian tumour tissues collected from the mice that had been treated with placental micro-EVs was examined. The fluorescence intensity of NKp46 was positively correlated to the fluorescence intensity of CD169^+^ macrophages in individual ovarian tumour collected from the mice that had been treated treated by placental micro-EVs (data not shown).

## Discussion

In this pilot *in vivo* study, we report that a single i.p. injection of placental EVs significantly inhibited the growth of SKOV-3 human tumour xenografts. Cellular necrosis was seen in ovarian tumour tissues, but not in other organs collected from mice treated with placental EVs. In addition, there was an extensive infiltration of CD169^+^ cells which are likely to be macrophages and NK cells into the ovarian tumours collected from mice that had been treated with placental micro-EVs. The increase in NK cells was positively correlated to the increase in CD169^+^ macrophages.

There is increasing evidence showing that extruded placental EVs are taken up by target cells and the EV cargo can then alter the function of target cells and/or organs which consequently regulates multiple maternal systems (reviewed in [24]). We have previously reported that a single tail vein injection of placental nano-EVs has significant impact on vascular function *in vivo* [25]. It has also been reported that EV-mediated intercellular communication is involved in cancer development and progression including immune suppression and metastasis (reviewed in [26–28]). In our current study, we found that a single ip injection of placental EVs significantly inhibited the growth of ovarian SKOV-3 tumour xenografts out to 58 days after injection. Although we have not investigated the distribution of placental EVs in our ovarian tumour model yet, a recent study showed that EVs extruded from the cells carry cellular ‘addressins’ on their surface which target the EVs to specific cell types, through specific pathways. For example, it has recently been shown that placental EVs are targeted to the lungs via RGD-containing proteins, while other placental EVs decorated with HYD-1 containing proteins, target the liver [29]. However, a current systematic review [30] showed that it is unlikely that EVs went to the grafted tumours, in particular in solid tumours, instead of spleen or liver. In addition, a recent study done by Minh Le from Singapore National University reported that EVs injected by the tail vein did not distribute to the tumour site in the mice model (these data were presented in 2023 Seattle ISEV meeting and the manuscript is under review). These findings suggested that there may be a different pathway in the distribution of EVs between organs and grafted tumours, although proteins expressed on the surface of the placental EVs, such as the calreticulin, ‘eat me’ signal protein [16] could guide the EVs targeting specific organs [29].

We have previously reported that placental EVs significantly inhibited the proliferation of ovarian cancer cells *in vitro* by delaying cell cycle progression [14]. Histological analyses of the placental EV-treated tumours in this current *in vivo* study (Figure 2) suggest that the tumours are undergoing necrosis [31,32]. However, there were no abnormal histological features in the liver and spleen tissues collected from the same mice that had been treated with placental EVs. This finding could further suggest that placental EVs may potentially be carrying molecules that interact selectively with the tumour tissues or alternatively, that the cargos of the EVs are only active when delivered to tumour cells. Recent study also reported that the delivery of cargos of the EVs contributed to the changes in the functions of target cells [33].

While the only markers of spontaneous necrosis are morphologic changes such as we report here, RIPK1 and MLKL are well-known mediators of programmed necrosis referred to as necroptosis [19]. When cells are undergoing death, RIPK1 is phosphorylated and forms an intracellular necrotic death complex. MLKL translocates to the plasma and organelle membranes and the subsequent permeabilization of these membranes leads to cell death [19,32,34–37]. We have previously reported that EVs from normal placentae contain MLKL and we wondered if phosphor-MLKL delivered to tumour cells via the EVs might have triggered the necroptosis pathway resulting in the necrosis of the tumours we saw [16]. However, there was no detectable expression of RIPK1 or phosphor-MLKL in our xenografted tumours that had been treated with placental EVs. Our data suggest that the RIPK1-MLKL associated death/necroptosis pathway may not be involved in the necrosis seen in our treated SKOV3 tumours. However, the necroptosis pathway is not the only trigger of necrosis [38]. In addition, the necrosis seen in ovarian tumour tissues could result from the secondary necrosis. Further study is required to understand the potential mechanism of causing necrosis in ovarian tumour tissues from placental EVs treated mice.

Placental EVs have been shown to have functions in modulating immune responses during pregnancy [39,40], suggesting that they may play a role in inducing immune responses or changing the immune environment. In addition, a most recent study reported that the delivery of miRNAs carried by EVs supressed breast cancer growth by triggering RIG-I medicated immune response [41]. In our current study, we found an increase in CD169^+^ that are likely to be macrophages in ovarian tumour tissues collected from mice that had been treated with placental micro-EVs. Although the SKOV-3 xenografts were implanted in immunodeficient mice, these animals are replete with both macrophages and NK cells. CD169^+^ is primarily expressed by macrophages although some other cells including myliod-derived dendritic cells may also express this marker. CD169^+^ macrophages are not typical M1/M2 macrophages. Rather they are normally located in the marginal zone of the spleen where they take up EVs from the blood [42]. CD169^+^ macrophages are also associated with anti-cancer immune responses with favorable clinical outcomes for cancer patients [20–22]. The increased presence of these macrophages in our xenografted tumours would suggest that they may play an important role in the mechanism(s) by which placental EVs inhibited/reduced the tumour growth.

EVs derived from dendritic cells can stimulate anti-tumour activities by NK cells [43], causing tumour regression [44]. Infiltration of NK cells was significantly increased in SKOV3 tumour tissues collected from mice that had been treated with placental micro-EVs, as measured by increased staining for NKp46, an activating receptor and marker of NK cells [45]. Increased infiltration of NK cells into tumours is generally associated with better cancer prognoses [46], and interestingly, we found a positive correlation between increased CD169^+^ macrophages and NKp46 cells in in our xenografted tumours collected from mice that had been treated with placental micro-EVs. Although further studies are required to confirm this, our results suggest that the regression of ovarian tumours in our study may be caused by the action of infiltrating CD169^+^ macrophages and NK cells.

The substantial infiltration of CD169^+^ macrophages and NK cells was only observed in ovarian tumour tissues collected from mice that had been treated with placental micro-EVs, not those mice treated with nano-EVs. Although we do not know the exact reason for this difference, previous studies reported that the cargos carried by micro- and nano-EVs are similar but with notable differences [47–49]. In addition, cells may interact with and process micro- and nano-EVs differently [50].

Placental EVs have been shown to carry many functional proteins, regulatory RNAs, DNA and lipids. We have yet to investigate which cargos contribute to the inhibition of SKOV3 tumour growth. However, our previous studies have found that placental EVs carry many proteins that regulate cell death [9–12], whilst a recent study also reported pregnancy-associated miRNAs having anti-cancer properties [15].

In this study placental micro- and nano-EVs had varying effects on regression of the ovarian tumours *in vivo*. While both types of EV reduced ovarian tumour growth relative to the control, with increasing time form the administration of the EVs, micro-EVs had a greater effect on tumour growth than the nano-EVs. We have previously reported that the cargos of placental micro- and nano-EVs are overlapping, but that each type of EVs also carry many components that are unique to either micro- or nano-EVs [16]. These unique components, most likely arise due to different biogenic pathways and functions of the micro- and nano-EVs and may account for the different effects of the two EV types on tumour regression in our model. Further investigation of the contents of both micro- and nano-EVs may shine light on the bioactives carried by these EVs that have anti-tumour properties.

There are several limitations in this pilot study. Firstly, the sample size was relatively small. Secondly, due to the efficacy, of particularly micro-EVs, in reducing tumour we were limited in the number of analyses we could perform on the tissues and in some cases the residual tumour was too small for analysis. Thirdly, we only examined the effect of anti-ovarian cancer growth by placental EVs in a single ovarian cancer cell line. Future, the xenograft models using multiple ovarian cancer cell lines, in particularly those high-grade ovarian cancer types should be performed.

In summary, in this pilot *in vivo* study, we found that a single intraperitoneal injection of EVs from normal placentae significantly inhibited growth in SKOV-3 ovarian tumour xenografts in immune-deficient nude mice. The growth inhibition was due cellular necrosis which may have been induced by CD169^+^ macrophages and NK cells that had abundantly infiltrated into the tumour tissues.

## Supplementary Material

Supplementary Figures S1-S8

## Data Availability

The datasets used and/or analysed during the current study are available from the corresponding author on reasonable request.

## Open Access

Open access for this article was enabled by the participation of The University of Auckland in an all-inclusive *Read & Publish* agreement with Portland Press and the Biochemical Society under a transformative agreement with CAUL.

## Abbreviations

C19MC: chromosome 19 microRNA cluster
EV: extracellular vesicle
MLKL: mixed linkage kinase domain-like
NK: natural killer
RIPK1: receptor-interacting kinase 1
