# Supplementary material for: A pilot in vivo study: potential ovarian cancer therapeutic by placental extracellular vesicles
Source: Biosci Rep. 2023 Aug 18;43(8):BSR20230307. doi: 10.1042/BSR20230307 (PMC10442519; doi:10.1042/BSR20230307)
Supplement: Supplementary Figures S1-S8 [file BSR-2023-0307_supp.pdf]

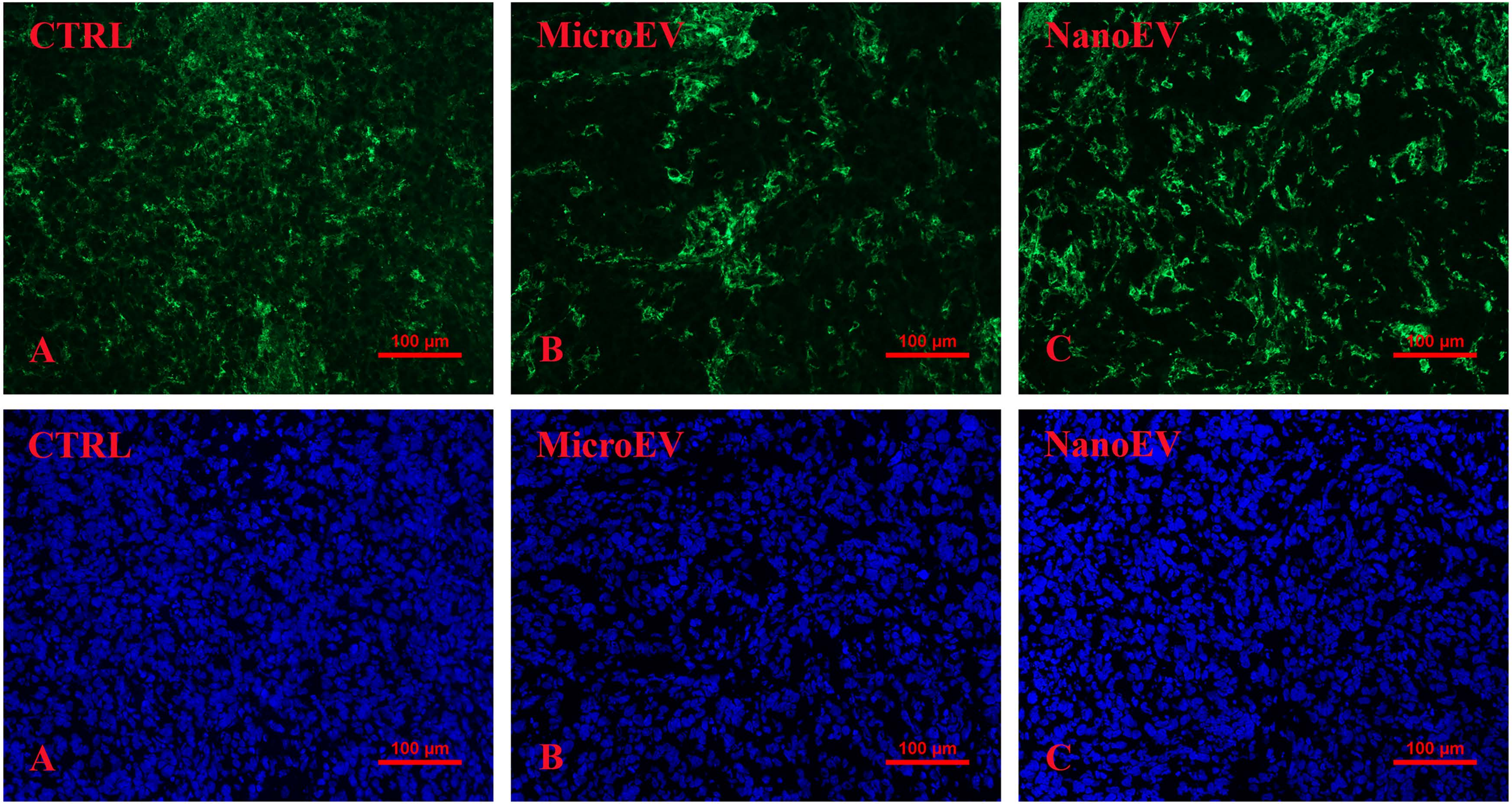

Figure 1 - Intensity of CD45 in tumours

There was no difference in the intensity of CD45 in tumours collected from mice that had been treated with placental micro-EVs (B), or placental nano-EVs (C), compared to untreated mice (A). DAPI was used for nuclear stains.

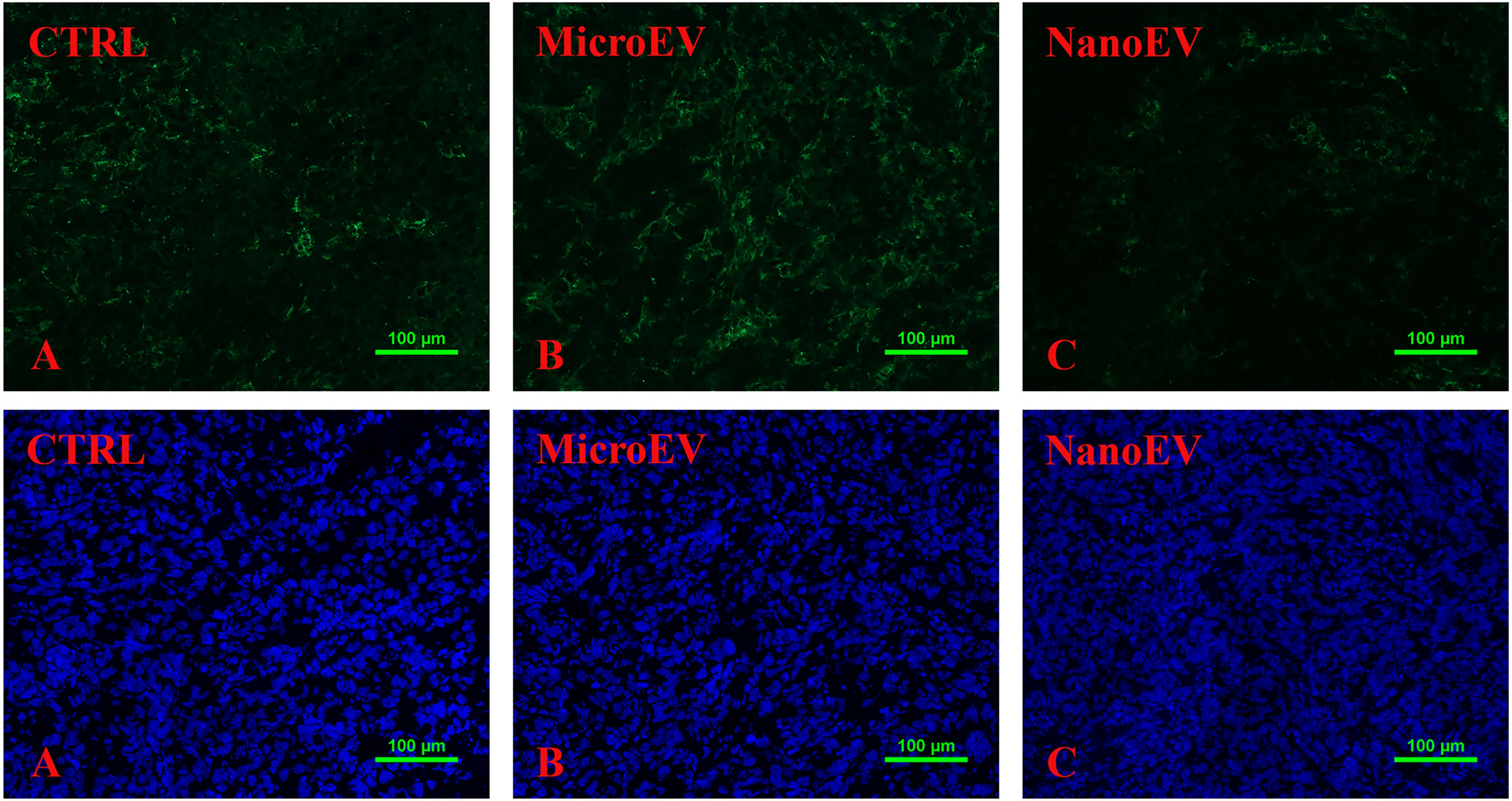

Figure 2 - Intensity of F4/80 in tumours

There was no difference in the intensity of F4/80 in tumours collected from mice that had been treated with placental micro-EVs (B), or placental nano-EVs (C), compared to untreated mice (A). DAPI was used for nuclear stains.

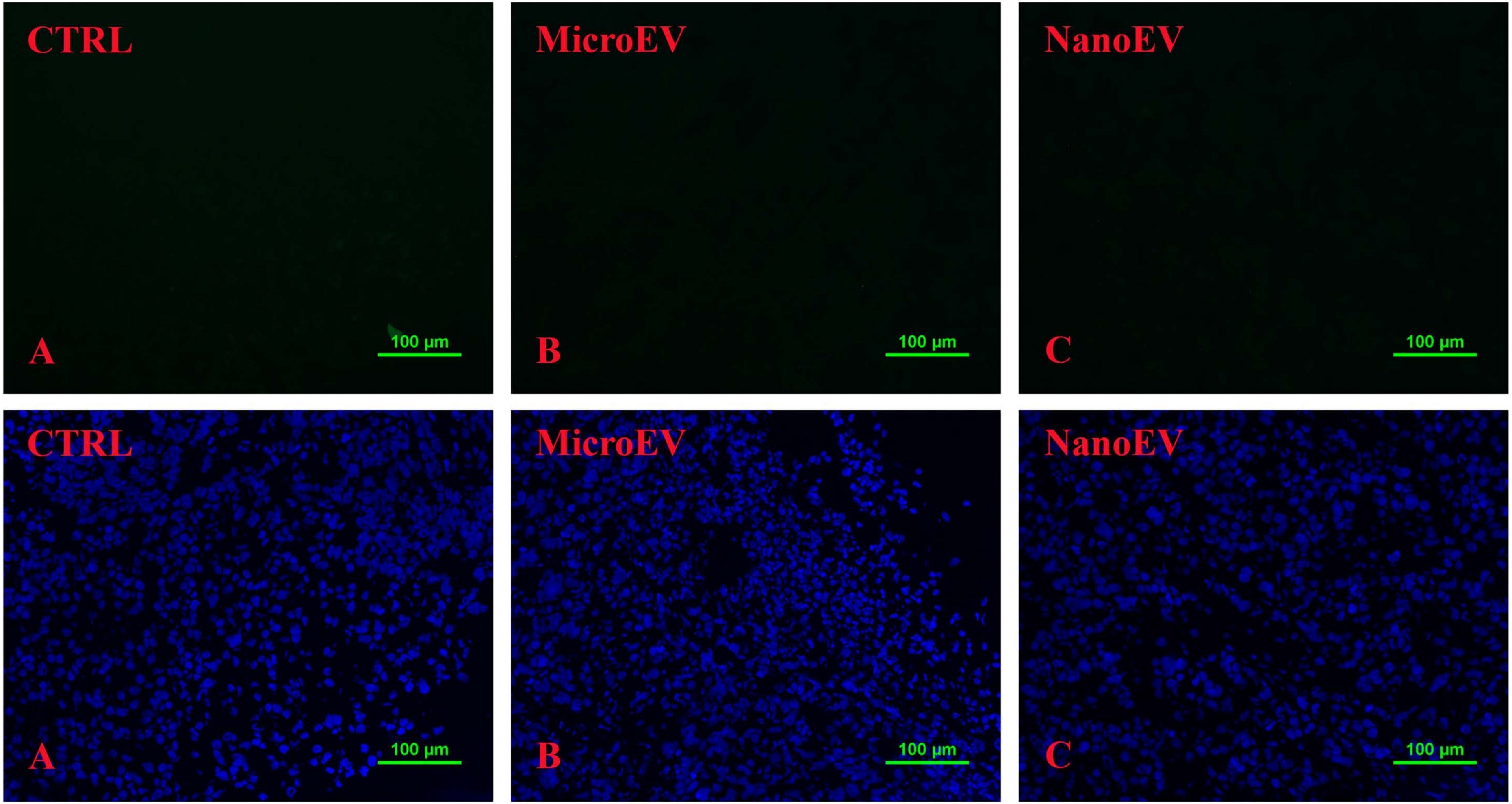

Figure 3 - Intensity of CD19 in tumours

There was no detectable intensity of CD19 in tumours collected from mice that had been treated with placental micro-EVs (B), or placental nano-EVs (C), compared to untreated mice (A). DAPI was used for nuclear stains.

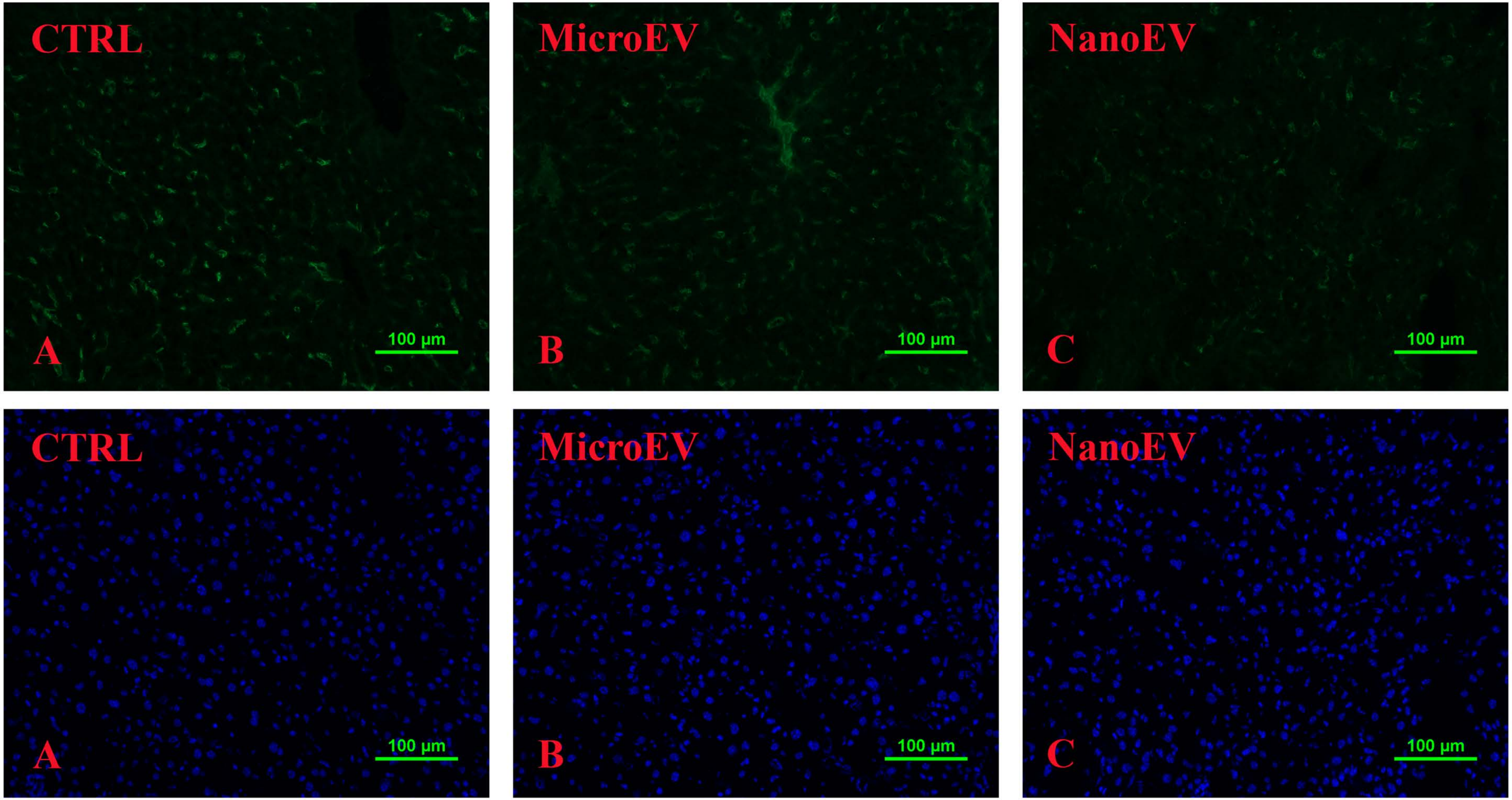

Figure 4 - Intensity of CD169+ macrophages in liver

There was no significant difference in the fluorescence intensity of CD169+ macrophages in the liver tissue collection from mice that had been treated with placental micro-EVs (A) and placental nano-EVs (B), compared to untreated mice (C). DAPI was used for nuclear stains.

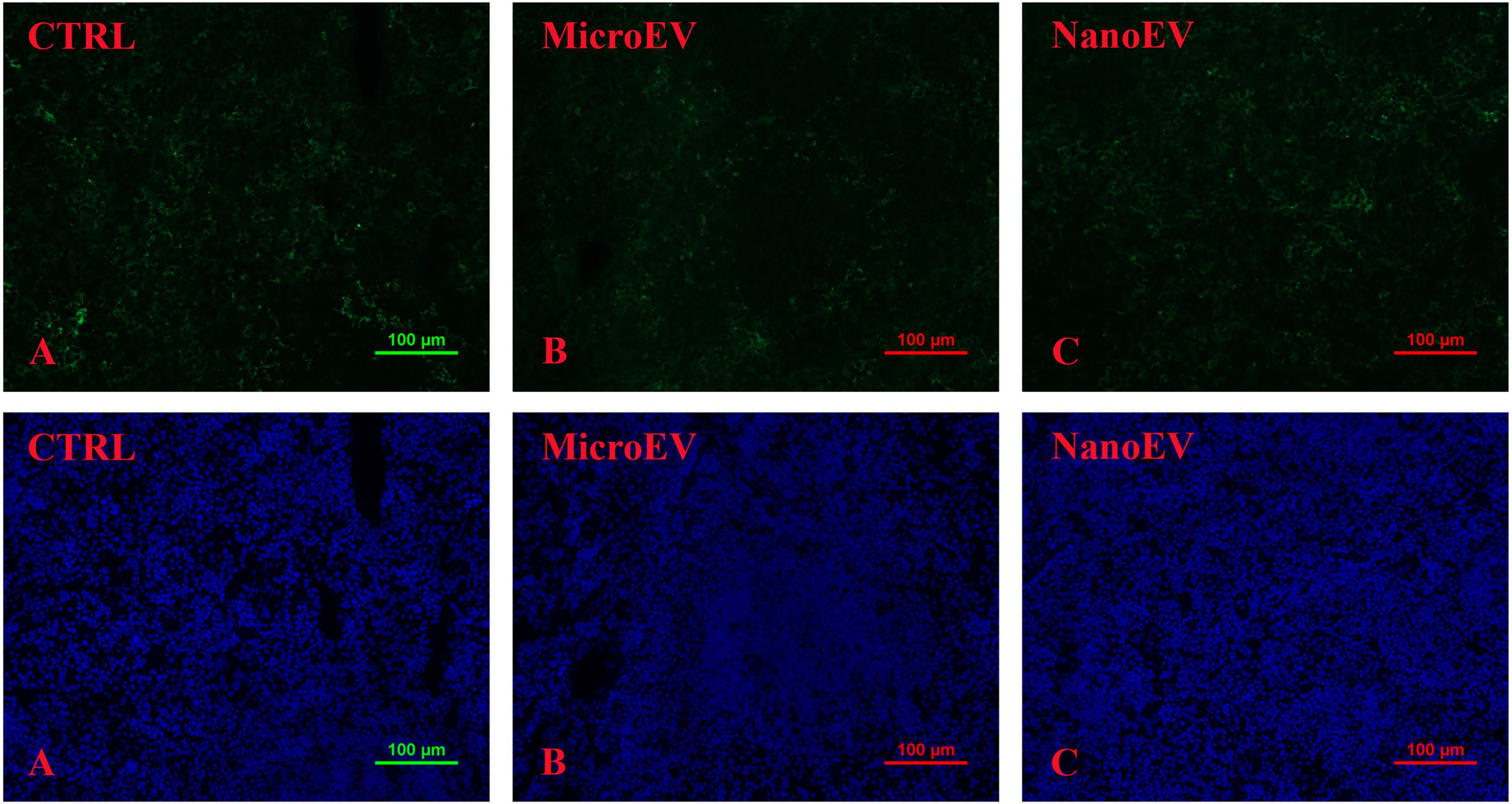

Figure 5 - Intensity of CD169+ in spleen

There was no significant difference in the fluorescence intensity of CD169+ macrophages in the spleen tissue collection from mice that had been treated with placental micro-EVs (A) and placental nano-EVs (B), compared to untreated mice (C). DAPI was used for nuclear stains.

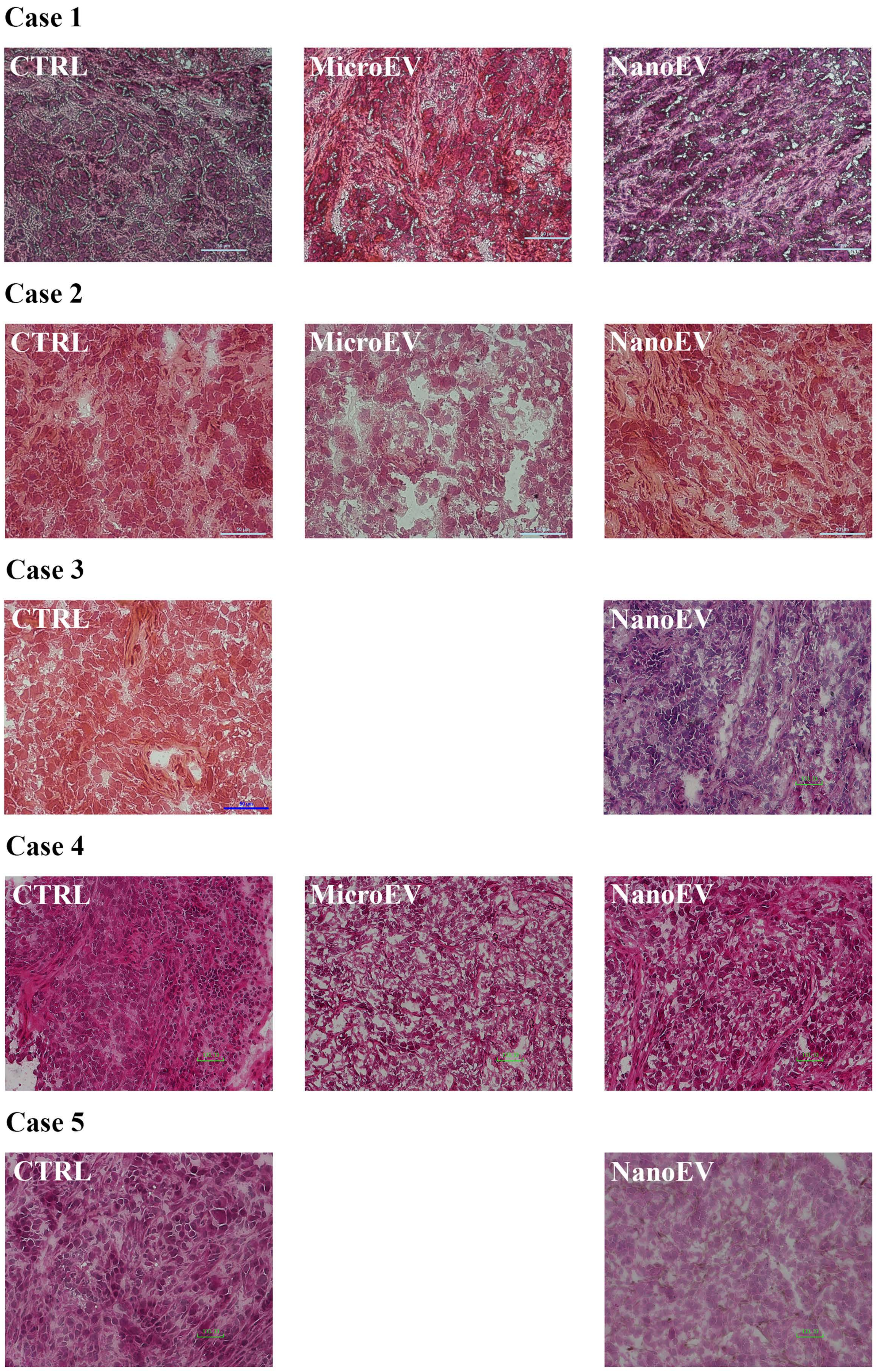

Figure 6 - H&E staining of tumours from each case

Signs of necrosis in tumors collected from individual treated mice by H&E stains

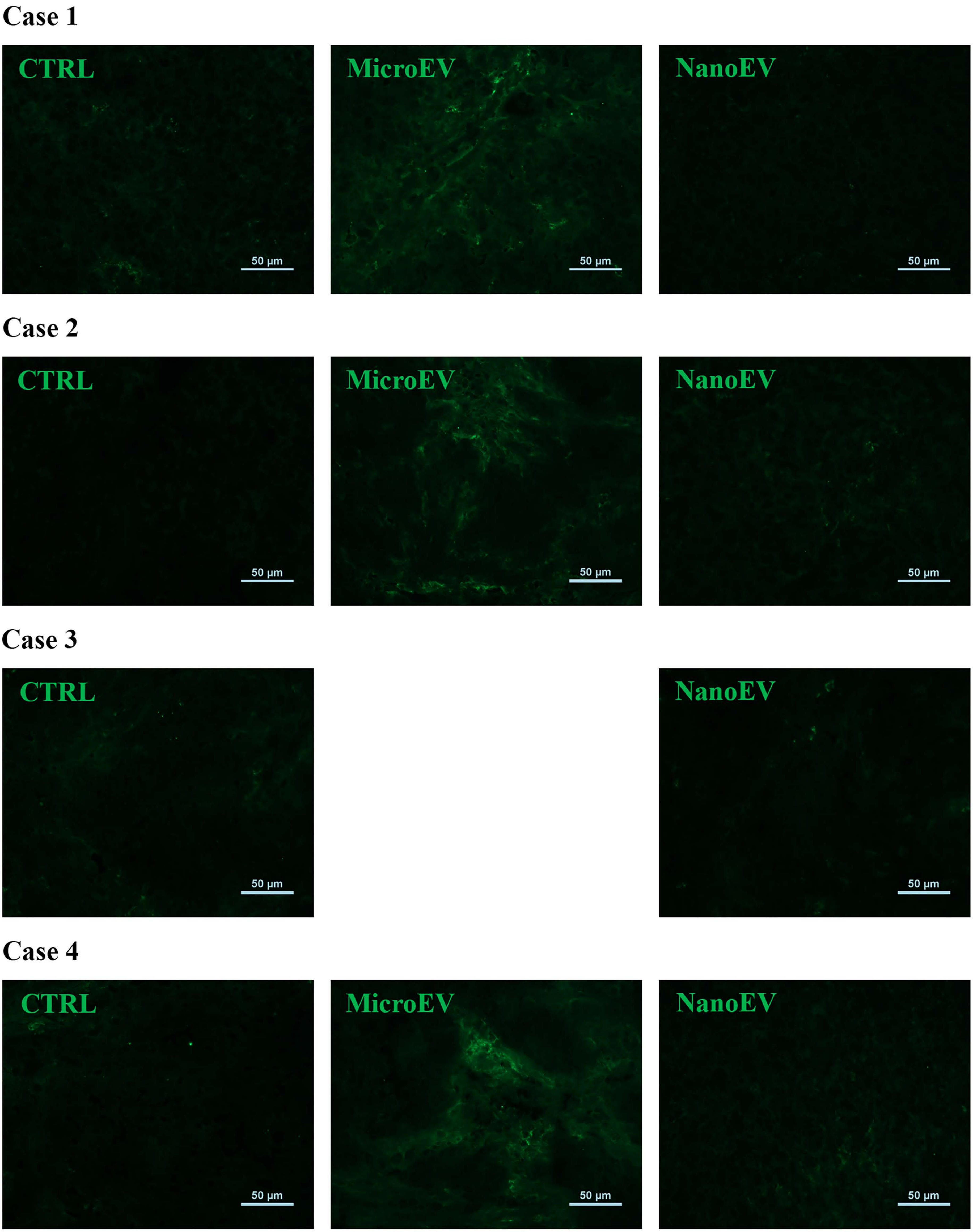

Figure 7 - Immunofluorescence staining of CD169+ macrophages in the tumours from each case

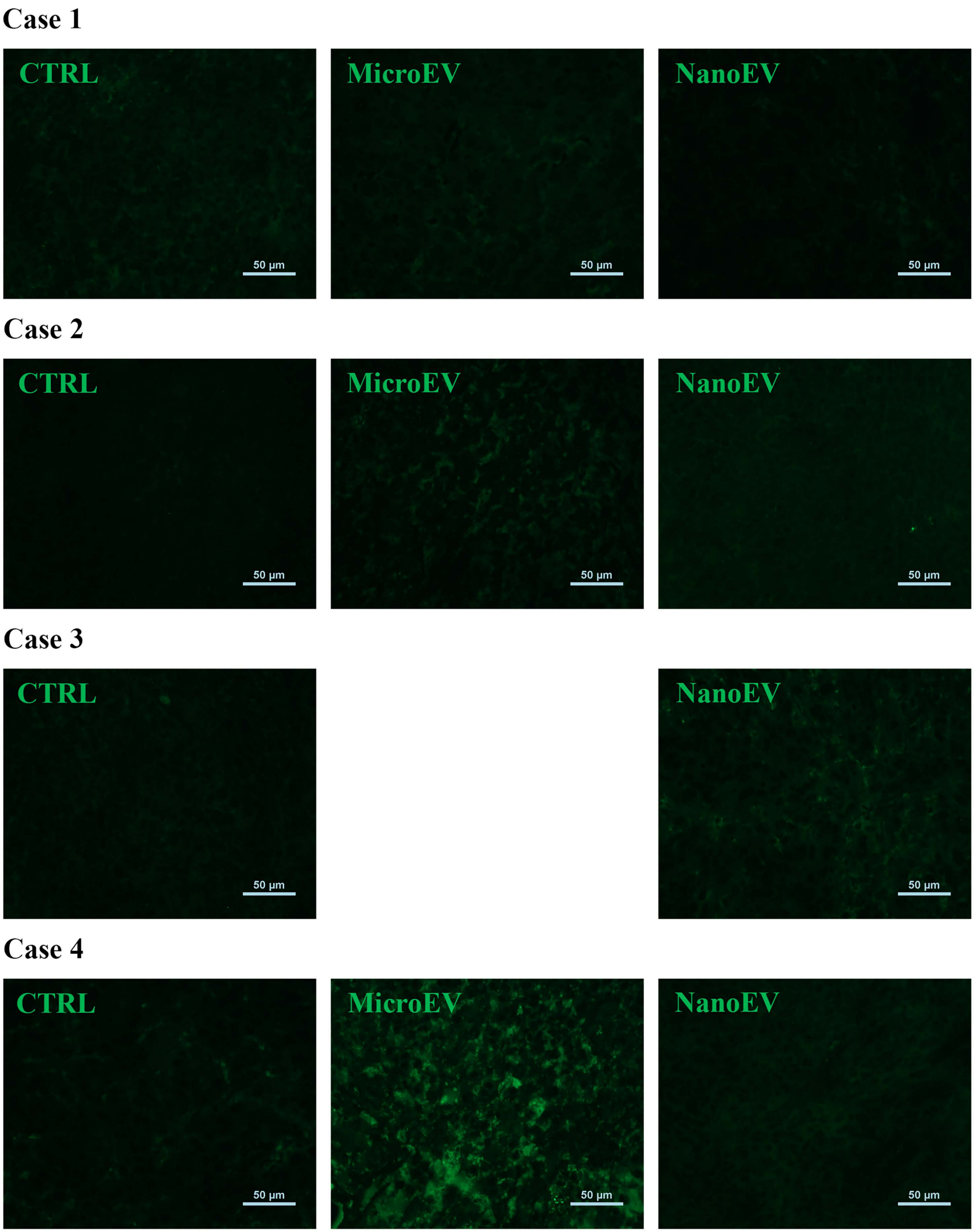

Figure 8 - Immunofluorescence staining of NK cells in tumours from each case
